# Application of volumetric absorptive microsampling (VAMS) for the simultaneous determination of cadmium and lead in blood

**DOI:** 10.1007/s10661-026-15796-y

**Published:** 2026-09-01

**Authors:** Claus Gutknecht, Alexandra Burianova, Stephan Bose-O’Reilly, Stefan Rakete

**Affiliations:** 1 Institute and Clinic for Occupational, Social and Environmental Medicine, LMU University Hospital, LMU Medicine, LMU Munich, Ziemssenstraße 1, 80336 Munich, Germany; 2 Institute of Public Health, Medical Decision Making and Health Technology Assessment, Department of Public Health, Health Services Research and Health Technology Assessment, UMIT - Private University for Health Sciences, Medical Informatics and Technology, Hall in Tirol, Austria

**Keywords:** Toxic metals, Human biomonitoring, Microsampling, ICP-MS, Background correction

## Abstract

Cadmium (Cd) and lead (Pb) are toxic elements. This study evaluates the applicability of volumetric absorptive microsampling (VAMS) as an alternative sampling method for biomonitoring of Cd and Pb in human blood. VAMS and blood samples were collected from 60 participants using untreated VAMS. Furthermore, samples from additional 27 participants were collected using VAMS pre-cleaned with 0.5% (*v*/*v*) nitric acid to reduce background levels in VAMS and improve performance. All samples were extracted (VAMS) or diluted (venous blood) with 0.5% (*v*/*v*) nitric acid prior to analysis by ICP-MS. For untreated VAMS, geometric mean (GM) recovery was 106.2% for Cd and 102.8% for Pb. For pre-cleaned VAMS, GM recovery was 117.2% for Cd and 104.6% for Pb. A strong correlation (Spearman Rho) between both methods was found (Cd, *ρ* = 0.84; Pb, *ρ* = 0.93). Correlation improved (Cd, *ρ* = 0.91; Pb, *ρ* = 0.98) for pre-cleaned VAMS. For untreated VAMS, the limits of quantification (LOQ) were 0.42 µg/l for Cd and 15.3 µg/l Pb. Pre-cleaning of VAMS led to a 90% background reduction for Pb (0.35 µg/l vs 3.61 µg/l). Background of Cd was unaffected by pre-cleaning (0.05 µg/l vs 0.07 µg/l). LOQs for pre-cleaned VAMS were 0.45 µg/l for Cd and 2.10 µg/l for Pb. Despite challenges like the background, VAMS is a promising alternative sampling method for human biomonitoring of Cd and Pb in blood. This study demonstrates the benefits of washing VAMS to reduce background.

**Supplementary Information:**

The online version contains supplementary material available at https://doi.org/10.1007/s10661-026-15796-y.

## Introduction

Cadmium (Cd) and lead (Pb) are toxic to plants, microorganisms, and animals, including humans. A wide range of effects and diseases are associated with exposure to these metals (Genchi et al., 2020; Tempowski & World Health Organization, 2021; WHO, 2019). Both elements are classified as persistent environmental pollutants as they are not metabolized or transformed into less toxic forms and bioaccumulate (UNEP, 2010; WHO, 2019). Traces of Cd and Pb can be found almost anywhere in the environment.

Both metals accumulate in the body, e.g., in tissue and bone. An exposure assessment via blood is suitable to reflect recent exposure from exogenous sources (Adams & Newcomb, 2014; Tempowski & World Health Organization, 2021). Venipuncture is the gold standard blood sampling method for the determination of Cd and Pb in blood (Adams & Newcomb, 2014; Breton et al., 2023). However, this sampling method bears relatively high logistical costs as it requires medically trained personnel. This may be a challenge for conducting human biomonitoring in remote or structurally weak regions (Basu et al., 2017; Guerra Valero et al., 2018; Jacobson et al., 2022; Lehner et al., 2013; Schweizer et al., 2021).

Microsampling methods in contrast offer a less complex and minimally invasive alternative (Williams & McDade, 2009). Rather small blood volumes (10 to 50 µl) are taken by finger or heel prick with a lancet. The capillary blood is blotted on filter paper (Dried Blood Spots, DBS) or wicked on special microsampling devices (e.g., volumetric absorptive microsampling, VAMS). After drying, samples can be packed for storage at ambient temperatures. Microsampling can be performed by non-medically trained personnel and is potentially suitable for the self-collection of samples (Allen et al., 2018; Sullivan et al., 2020). In addition, other studies show a very high acceptability and willingness of participants to provide microsamples (Andrew et al., 2022; Williams & McDade, 2009), which is essential for biomonitoring studies or for monitoring purposes in occupational settings.

DBS sampling is widely used in numerous applications, including the potential use in biomonitoring for some heavy metals in blood, as Schweizer demonstrated for mercury (Hg) (2021). Studies that used DBS for the quantification of Cd and Pb levels in blood found background levels in the sampling material that exceeded the blood levels of non-exposed participants in the analyte (Chaudhuri et al., 2009; Funk et al., 2013). Therefore, quantitation of Pb and Cd was not possible. A second drawback of this method is the hematocrit dependency, meaning the hematocrit content, and thus the blood viscosity, affects the blood volume per surface area absorbed by the filter paper and consequently the amount analyzed for quantification (Breton et al., 2023; Chaudhuri et al., 2009; Demirev, 2013; Spooner et al., 2015).

The VAMS method on the other hand promises an accurate volume sample collection, independent of the hematocrit (Protti et al., 2019; Spooner et al., 2015). The VAMS method uses hydrophilic polymer tips, attached to a plastic handle, which allows a rather easy and convenient sample collection (Denniff & Spooner, 2014; Spooner et al., 2015). The tip absorbs a fixed volume of blood by wicking. Until recently, VAMS application has mainly been used in monitoring drug molecules, analyzing peptides and proteins, forensic analysis of drugs, syndrome screening, and anti-doping testing in blood and other specimens (Protti et al., 2019, 2021; Verstraete & Stove, 2021).

To our knowledge, VAMS has only been used in a limited number of studies for trace metal analysis in human blood. It has been used to quantify prosthesis-related metals (Al, Ti, V, Co, Cr, Ni, Sr, and Zr) (Bolea-Fernandez et al., 2016), Hg (Koutsimpani-Wagner et al., 2022), Pb (Breton et al., 2023) and recently a wider range of essential and toxic trace elements (Al, As, Cd, Cr, Co, Cu, Hg, Mn, Mo, Pb, Se, U, Zn) (Breton et al., 2024). Another study explored its potential using VAMS devices spiked with reference materials (As, Be, Cd, Cs, Cu, Fe, Mg, P, Pb, S, Sb, Se, Tl, V, and U) (Schmidt et al., 2024). Although VAMS has been used for the simultaneous biomonitoring of Cd and Pb under field conditions in one study (Breton et al., 2024), relatively high LOQs remain problematic. The goal of this study was the development and validation of a biomonitoring method for the simultaneous determination of Cd and Pb using VAMS in combination with ICP-MS analysis as well as exploring the potential of pre-cleaning VAMS prior to use.

## Materials and methods

### Materials and reagents

Nitric acid Optima™ (67–69%) for ultratrace elemental analysis was obtained from Fisher Scientific (Leicestershire, UK). Ultrapure water (resistivity > 18.2 MΩcm) was provided from a Direct-Q® 3 UV Water Purification System from Merk Millipore (Darmstadt, Germany). ICP-MS Stock Tuning Solution, Internal Standard Mix containing 10 mg/l Tb and Environmental Calibration Standard containing each 10 mg/l Cd and Pb were obtained from Agilent (Santa Cruz, USA).

**Fig. 1:**
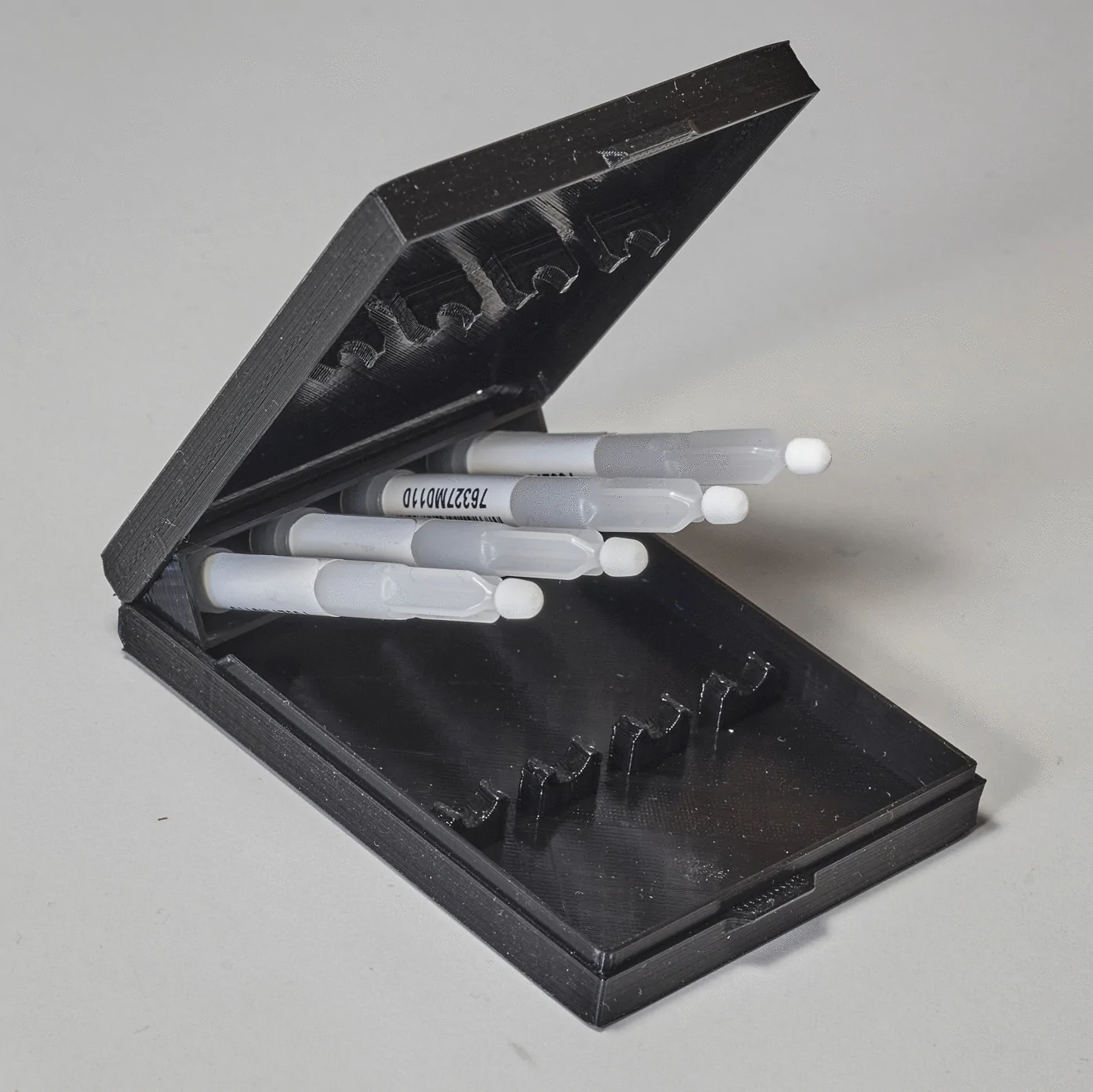
VAMS devices in the 3D-printed holder

Quality control material (QC) for whole blood (ClinCheck®, Level I, 1.58 µg/l Cd; 37.6 µg/l Pb) was obtained from RECIPE (Munich, Germany). VAMS devices (Mitra™) were obtained from Neoteryx LLC (Torrance, USA) and consisted of 4 VAMS tips per holder (clamshell). Three different VAMS batches were used for the study: 54 from LOT 74918, six from LOT 76327, and 27 from LOT 95222). VAMS sampling volumes were 23.6 µl (LOT 74918 and LOT 76327) and 24.6 µl (LOT 95222), respectively. Original clamshells were replaced by custom-made, 3D-printed clamshells (Fig. 1) made from polylactide (PLA) filaments, obtained from Form Futura (Nijmegen, Netherlands).

Venous blood was collected into Li-Heparin-coated S-Monovette® 7.5 mL tubes for trace metal analysis (Sarstedt®) by venipuncture. For fingerpricking, disposable lancets (Solofix) from B. Braun (Melsungen, Germany) were used. Clamshells containing the blood samples were stored at room temperature in aluminum-coated mylar ziplock bags (12 × 20 cm) until analysis.

### Study design

This study was reviewed by the ethics committee of the Ludwig Maximilians University of Munich (#20–091) and conducted according to The Code of Ethics of the Declaration of Helsinki for human experiments. All participants signed an informed consent form prior to sampling and were asked to fill out a questionnaire about possible Cd and Pb exposure. Paired venous and capillary blood samples were collected from consenting, occupationally non-exposed, adult human subjects at the Clinic for Occupational, Social and Environmental Medicine, University Hospital, LMU Munich. Sixty paired VAMS and venous blood samples were collected in January and February 2023. Untreated VAMS devices were used as supplied by the manufacturer. Additionally, 27 paired VAMS and blood samples were collected from December 2023 to January 2024 using VAMS devices that had been pre-cleaned to reduce background levels of Cd and Pb.

### Sample collection

Approximately 7 mL of blood was collected from each participant by venipuncture. Venous blood samples were stored at −20 °C until analysis. VAMS sampling devices were prepared in batches of four per clamshell. For each participant, an individually prepared clamshell was used. After fingerpricking, three sampling devices were used to collect three separate capillary blood samples per subject. The fourth device was left empty to quantify background levels of Cd and Pb. To avoid sample contamination, the capillary blood collection followed a strict protocol. Participants were asked to thoroughly wash their hands and dry them with disposable paper towels. The finger was disinfected and kept, and the disinfectant was allowed to air-dry. Pricking was done in the fingertip with a disposable lancet. The first drop of blood was wiped away with sterile gauze. If necessary, blood flow was stimulated by gently massaging the finger towards the tip, creating a blood stasis, without applying too much pressure next to the pricked area to avoid sample dilution by tissue fluid. In some cases, and with consent of the participants, a second prick was performed for sufficient blood collection. To ensure a reliable blood volume collected by VAMS, the polymer tips were just slightly touching the blood drop at the finger without submerging it as recommended by the manufacturer (Neoteryx, 2022). Subsequently, the samples were placed upside down within the slightly opened clamshell in a desiccator and dried for at least 2 h. Finally, the clamshells were packed in aluminum-coated mylar ziplock bags, which were additionally thermally sealed at the site of collection. VAMS samples were stored at room temperature until analysis. The storage time varied between 14 and 42 days for untreated VAMS and 14 to 51 days for pre-cleaned VAMS.

### Pre-cleaning of VAMS

When using VAMS as supplied by the manufacturer, relatively high background levels of Cd and Pb were observed. Thus, VAMS devices were washed in 0.5% (*v*/*v*) nitric acid in an ultrasonic bath for 45 min at 60 °C and subsequently rinsed in ultrapure water for 15 min under the same conditions. VAMS devices were washed upside down, with their tips submerged in the washing solution and subsequently dried overnight in a desiccator. The VAMS devices were then used as described above to collect blood samples. Potential changes in the blood wicking capability due to the pretreatment were assessed using eight pre-cleaned and eight untreated VAMS. This assessment involved weighing the devices individually before and immediately after sampling capillary blood from the same person.

### Instrumentation

For sample analysis, an 8900 Triple Quadrupole ICP-MS equipped with an Integrated Autosampler (I-AS) from Agilent (Santa Cruz, USA) was used. The instrument was tuned daily to achieve optimum sensitivity, oxide ratio, and doubly charged ratio. The ICP-MS operating conditions were as follows: RF power, 1600 W; plasma gas flow, 15 l/min; nebulizer gas flow, 0.82 l/min (venous blood) or 1.05 l/min (VAMS); auxiliary gas flow, 0.9 l/min; dilution gas, 0.15 mL/min (only venous blood); replicates, 3; sweeps/replicate, 30. All analyses were run in single quadrupole mode using helium as collision gas (5.5 mL/min) to reduce polyatomic interferences. ^111^Cd and ^206+207+208^Pb were quantified using an external calibration. As internal standard, ^159^ Tb was used.

### Sample preparation

Prior to the analysis, venous blood samples were thawed on a roll mixer. Analysis was carried out in triplicate. Three aliquots of 50 µl were diluted 20-fold with 0.5% (*v*/*v*) nitric acid containing 10 µg/l Tb as internal standard in 2-mL polystyrene sample cups obtained from WICOM Germany (Heppenheim, Germany) and analyzed by ICP-MS. For quality assurance, QC samples were treated as regular blood samples analyzed at the beginning of every sample batch.

For VAMS analysis, individual polymer tips (three blood samples and one blank per participant) were placed each in a separate 2 mL safe-lock tubes (Eppendorf, Hamburg, Germany). To avoid contamination, the VAMS tip was removed from the holder by gently pinching the tip with the tube’s lid and simultaneously pulling the holder. For extraction, 700 µl 0.5% (*v*/*v*) nitric acid containing 10 µg/l Tb as internal standard was added to the tube. The samples were then extracted in an ultrasonic bath (Sonorex Super RK 103 H from Bandelin, Berlin, Germany) for 45 min at 40 °C and subsequently centrifuged at 16,000 g for 5 min using a Galaxy 16 DH from VWR (Darmstadt, Germany). A 650 µL aliquot of the supernatant was transferred into a 2-mL polystyrene sample cup for ICP-MS analysis. For quality assurance, QC samples were pipetted onto VAMS tips and treated like regular VAMS blood samples and analyzed at the beginning of every sample batch.

### Statistical analysis and data processing

Initial data processing was carried out using Microsoft Excel (Microsoft Office LTSC Professional Plus 2024). Statistical analysis and graph preparation were performed using RStudio 2025.05.1 + 513 “Mariposa Orchid” Release for Windows, utilizing R version 4.5.1.

Prior to the statistical analysis, all six VAMS samples from LOT 76327 were excluded due to high and unevenly distributed Cd levels (supplementary material). Two VAMS samples from LOT 76327 and one VAMS sample from LOT 95222 were excluded due to a handling mistake during the sampling, i.e., touching of tip on a surface (supplementary material). Thus, data from 52 participants were used for untreated VAMS and from 26 participants for pre-cleaned VAMS (this data is available as supplementary material).

Venous blood levels of Cd and Pb were calculated as the mean of the three independent replicates per participant and represent the reference blood Cd and Pb levels (gold standard method). VAMS Cd and Pb levels were in principle calculated similarly. To address potential contamination and/or undersampling of individual VAMS tips, an exclusion criterion was used. In detail, the VAMS replicate with the largest difference from the mean was excluded if the relative standard deviation (RSD) exceeded 30%.

Due to the background levels of Cd and Pb in VAMS devices, a background correction using the mean background level for each element and experiment (untreated and pre-cleaned VAMS) was performed. Prior to the calculation of the mean background level, extreme values exceeding a predefined threshold (75th percentile + 1.5* interquartile range) were excluded (supplementary material). For untreated VAMS, 49 Cd blanks and 47 Pb blanks were included. For pre-cleaned VAMS, 25 Cd blanks and 22 Pb blanks were included (see supplementary material). Finally, the resulting mean background level was subtracted from the individual mean VAMS level of every participant.

The limits of detection (LOD) and limits of quantitation (LOQ) were calculated as three times and ten times the standard deviation (SD) of the blank levels for each element and experiment, respectively. The recovery was used as a measure of accuracy, calculated from the corresponding VAMS and venous blood samples ($recovery \left[\%\right]=\frac{{c}_{VAMS}}{{c}_{VB}}*100 \%).$ The RSD of VAMS replicates was used as a measure for precision. For correlation analysis of venous blood and VAMS samples, Spearman Rho and Passing-Bablok regressions were used. Correlation results are graphically displayed as Passing-Bablok regression graphs. Difference plots were used to visualize the differences between both methods. Storage stability was tested by a linear model (storage time ~ recovery). The effect of pre-cleaning VAMS devices on the wicking capacity was tested using a two-sided *t*-test.

## Results and discussion

### Venous blood results

The venous blood Cd levels from all participants ranged from 0.06 to 1.34 µg/l. All samples were above the methods LOD of 0.03 µg/l and 74 samples (95%) were above the methods LOQ of 0.1 µg/l. The mean, median and 95th percentile for Cd were 0.34 µg/l, 0.24 µg/l, and 1.15 µg/l, respectively. For Pb, the venous blood levels ranged from 4.57 to 49.77 µg/l. All samples were above the methods LOQ of 0.33 µg/l. The mean, median, and 95th percentile for Pb were 11.70 µg/l, 9.86 µg/l, and 20.67 µg/l, respectively. Mean recoveries for QC samples were 94% for Cd and 97% for Pb, respectively.

The Cd levels found were comparable to what has been reported for non-smokers in the German Environmental Survey III (GerES III) from 1998 (Becker et al., 2002). However, the 95th percentile in this study was higher, suggesting that some of the participants were smokers. However, the participants did not provide information on their smoking status. Thus, higher Cd may also be explained by other factors such as diet. In contrast, the Pb levels found in this study were much lower compared to the results of GerES III, likely due to the phase-out of leaded gasoline. In fact, the Human Biomonitoring Commission of the German Environment Agency reported comparable Pb levels among students in Germany between 2010 and 2015 (Kommission Human-Biomonitoring des Umweltbundesamtes, 2019). Therefore, the participants can be considered to be low exposed individuals.

### VAMS background levels

Inherent background levels are one of the biggest concerns when microsampling devices such as DBS or VAMS are applied to human biomonitoring of trace elements. In this study, high VAMS background levels equivalent to up to 373 µg/l were observed for Pb. A comparison of blank levels from other studies using VAMS can be found in Table 1. Furthermore, background levels were relatively unevenly distributed and batch-dependent. In this study, mean blank correction was found to superior compared to blank correction using the individual blank of each participant. Nevertheless, the relatively high uncertainty of blank levels, expressed as variance, limits the method’s potential. In this study, the blank level variance was used for LOD and LOQ calculation, as this effect is predominant compared to the influence of the blood matrix. As shown in Table 1, Cd blank levels and variance as well as LOD and LOQ were nearly unaffected by pre-cleaning of VAMS. The diagnostic sensitivity was comparable to what has been found in other studies (Breton et al., 2024; Schmidt et al., 2024). In contrast, the mean Pb blank level was reduced by more than 90% by pre-cleaning of VAMS. Furthermore, the variance of the blank levels was reduced by more than 85%. This led to a significant improvement of the diagnostic sensitivity for Pb, which was superior to other studies using VAMS (Breton et al., 2023, 2024; Schmidt et al., 2024). The wicking capacity of VAMS was not significantly affected by the pre-cleaning procedure.

**Table 1:** VAMS blank levels of Cd and Pb in this study vs. other studies using VAMS (all results in µg/l)

	Cd				Pb			
	Mean	SD	LOD	LOQ	Mean	SD	LOD	LOQ
**This study, untreated**	0.067	0.042	0.13	0.42	3.61	1.53	4.60	15.32
**This study, pre-cleaned**	0.054	0.045	0.14	0.45	0.35	0.21	0.63	2.10
Breton et al. (2023)	NR	NR	NR	NR	NR	NR	1.66	5.59
Schmidt et al. (2024)	< 0.06	NR	0.18	0.60	3.03 −6.57	NR	3.06	10.1
Breton et al. (2024)	< 0.0001	NR	0.17	0.56	1.99	NR	4.35	14.50

*NR* not reported

### VAMS performance

The main objective of this study was to evaluate the applicability of VAMS as an alternative sampling tool for human biomonitoring of Cd and Pb in blood. After the first sampling series using untreated VAMS as supplied by the manufacturer, the background levels were identified as one of the major limitations for VAMS. Thus, a second sampling series was conducted after development of a cleaning protocol for VAMS.

The rationale of using an RSD of 30% as a cutoff for the exclusion of single VAMS replicates is suggested as a reasonable way to deal with some of the challenges of microsampling, e.g., unevenly distributed background levels and the possible contamination of samples during sampling as well as potential over- and undersampling. The latter can be avoided if VAMS is used according to the manufacturer’s instructions. With VAMS, it is not possible to repeat an analysis once they are extracted. Thus, the replicate with largest difference from mean was excluded when the predefined criterion was met. This was applied to 19 samples for Cd (36.5%) and 6 samples for Pb (11.5%) for untreated VAMS. For pre-cleaned VAMS, this was applied for five samples for Cd (19.2%) and one sample for Pb (3.7%). Therefore, pre-cleaning of VAMS led to an improvement regarding precision.

An overview of the VAMS performance parameters for QC samples can be found in Tables 2 and 3 for participant samples. The recovery as a measure of accuracy was a pivotal quality criterion. Mean recovery for QC samples using VAMS was 101% for Cd. For participant samples, both untreated and pre-cleaned VAMS showed satisfactory results for Cd in terms of recovery and precision, and about 70% of the samples were in the desired range of recovery (70 to 130%). All samples outside of this range were from participants with Cd levels in venous blood of less than 0.4 µg/l, thus lower than the calculated LOQ of Cd for VAMS. This suggests a certain degree of uncertainty, especially at low levels. Nevertheless, VAMS showed good recoveries even at Cd levels lower than 0.4 µg/l. Surprisingly, pre-cleaning of VAMS had little effect on the recovery and precision. This is likely due to the fact that Cd levels in VAMS are already low by default. Furthermore, more single replicates were excluded for untreated VAMS prior to the statistical analysis (see above). This has led to an alignment of the mean precision. The increased recovery rate for pre-cleaned VAMS may be attributed to batch-specific variations in the blood volumes absorbed by the material. However, this has not been tested.

**Table 2:** VAMS performance parameters for QC samples

	VAMS treatment	QC target mean (range)	QC VAMS mean ± SD
**Cd**	No	1.58 (1.19–1.98) µg/l	1.58 ± 0.12
	Yes		1.60 ± 0.07
**Pb**	No	37.6 (30.1–45.1) µg/l	37.1 ± 4.3
	Yes		34.6 ± 0.6

**Table 3:** VAMS performance parameters for participant samples

	VAMS treatment	GM recovery	recovery range	70% < recovery < 130%	Mean RSD	Spearman-Rho (ρ)	Samples > LOD	Samples > LOQ
**Cd**	No (52)	106.2%	36–692%	37 (71%)	14.0%	0.839	47 (90%)	13 (25%)
	Yes (26)	117.2%	54–1670%	18 (69%)	16.1%	0.913	24 (92%)	6 (23%)
**Pb**	No (52)	102.8%	70–146%	50 (96%)	8.72%	0.927	51 (98%)	7 (14%)
	Yes (26)	104.6%	90–226%	24 (92%)	7.62%	0.981	26 (100%)	26 (100%)

*GM* geometric mean

For Pb, VAMS performance was overall better compared to Cd, which can be explained by the generally higher levels of Pb in blood. Mean recovery for QC samples using VAMS was 96% for Pb. More than 90% of the participant samples were in the desired range of recovery (70 to 130%). The few samples outside the desired range of recovery had Pb level in venous blood of less than 10 µg/l. Again, most samples below 10 µg/l still showed a satisfactory recovery. As for Cd, pre-cleaning of VAMS had only little effect on the recovery and precision, even though the mean background was reduced dramatically. This suggests that even at relatively high background levels, the background correction using the mean background as well as the exclusion of single replicates is valid.

**Fig. 2:**
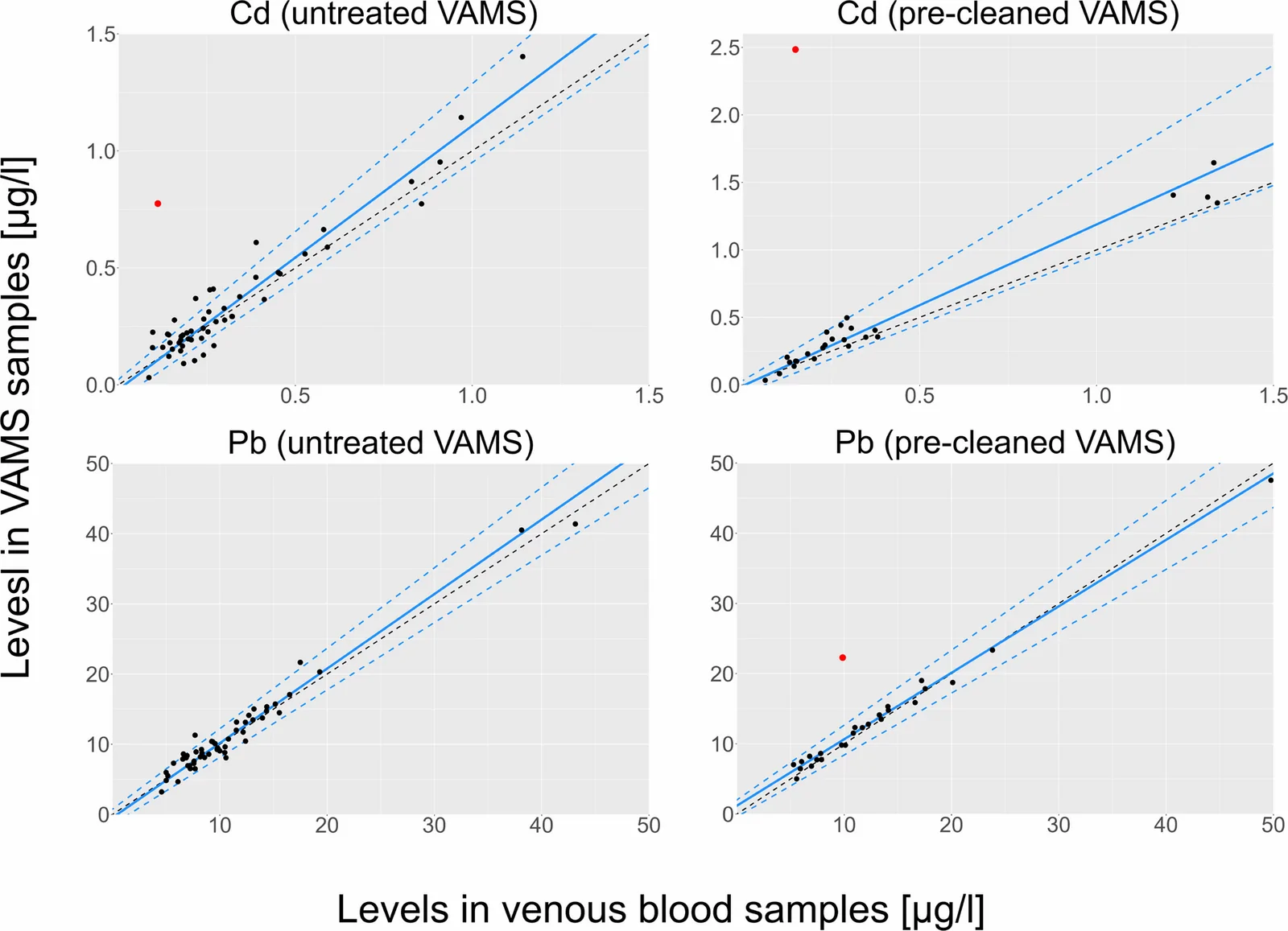
Passing-Bablok regression plot of Cd and Pb levels in venous blood vs. levels in corresponding VAMS samples. The light blue solid line represents the regression line, the light-blue dashed lines show the limits of the 95% confidence interval of the regression line. The dashed black line represents the identity line. Samples excluded from regression analysis are depicted as red dots

Venous blood levels of Cd and Pb showed excellent correlation with VAMS levels (Fig. 2). Prior to regression analysis, one conspicuous venous blood-VAMS pair for each Cd in untreated VAMS, Cd in pre-cleaned VAMS, and Pb in pre-cleaned VAMS was excluded. The deviation from the regression line can be explained by an unnoticed contamination of the VAMS tips during sampling or processing. Interestingly, the excluded untreated VAMS sample showed an extremely high recovery for Cd, but was unremarkable for Pb. In contrast, the excluded sample for pre-cleaned VAMS was conspicuous for both Cd and Pb. The data points were included in the regression plots to show that outliers like these can occur even if strict protocols for avoiding sample contaminations were used. However, even if these samples were included in the analysis the correlation would be still strong (Cd (untreated VAMS), *ρ* = 0.761; Cd (pre-cleaned VAMS), *ρ* = 0.777; Pb (pre-cleaned VAMS), *ρ* = 0.920).

As observed for recovery and precision, regression results were better for Pb than for Cd. The correlation results for Pb are comparable with those of Breton et al. (2023). Furthermore, the Passing-Bablok regression lines were almost overlapping with the identity line, confirming an excellent agreement of both methods. For Cd, the regression line had a steeper slope than the identity line. Therefore, VAMS seems to overestimate Cd levels in blood. However, the 95% confidence intervals of the intercepts encompass 0, implying the absence of systematic differences. Moreover, no proportional differences between the methods are evident, as the 95% confidence intervals for the slope of both elements encompass 1.

In contrast to recovery and precision, pre-cleaning of VAMS significantly improved the correlation between venous blood samples and VAMS. The scattering of the individual data points was less pronounced for pre-cleaned VAMS. Furthermore, all data points of the pre-cleaned VAMS were within the 95% confidence intervals of the Passing-Bablok regression. While this also true for most of the untreated VAMS, a few samples were outside the confidence intervals. Although pre-cleaning does on average not have a significant effect on recovery, it seems that the deviation from the gold standard decreased due to reduced background levels.

**Fig. 3:**
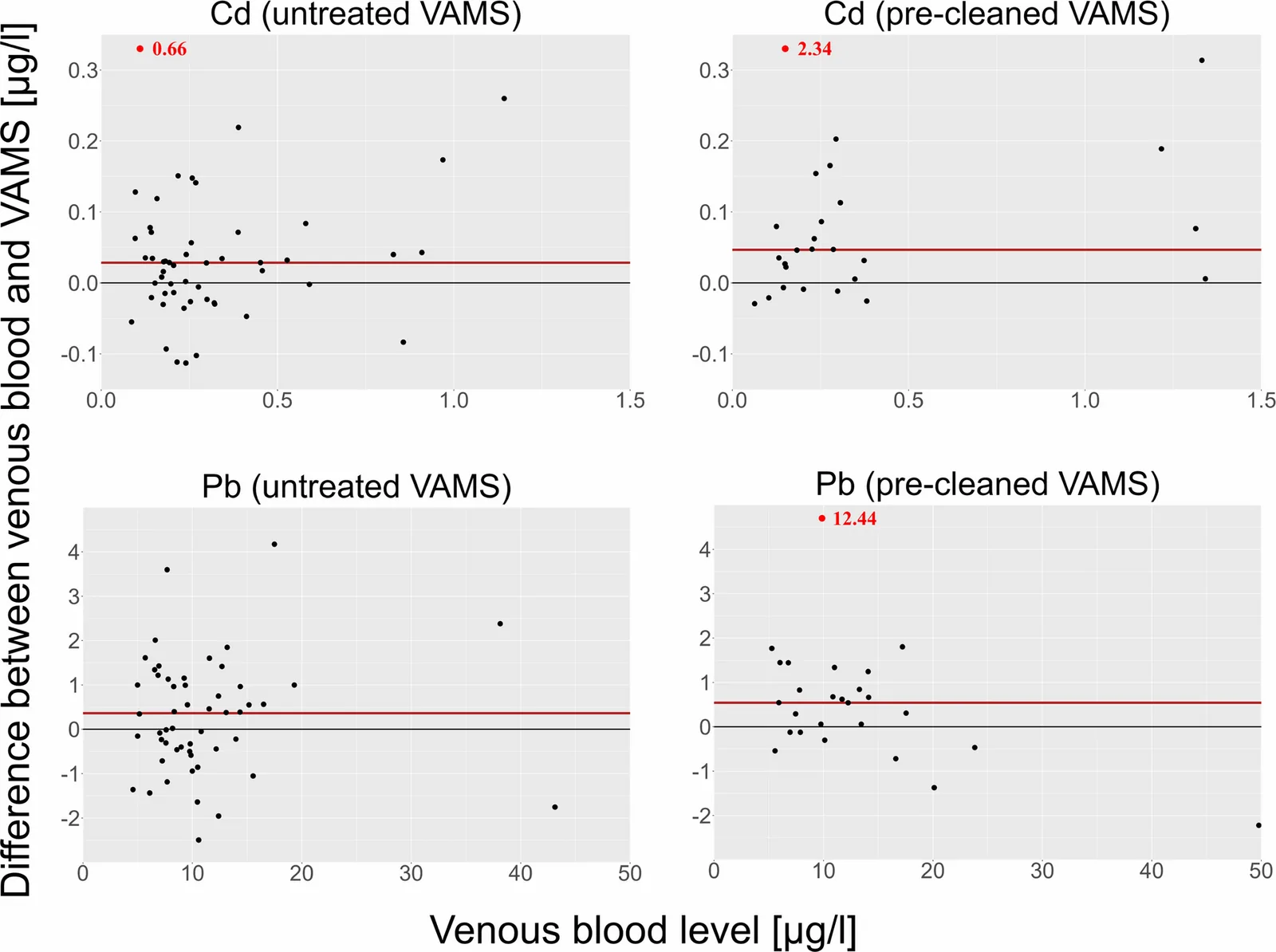
Difference plots showing the absolute differences between levels in venous blood and VAMS samples vs. the levels in venous blood samples. The median differences are shown as red line. Samples with a large difference were depicted as red dots to ensure readability of the graph

In Fig. 3, the absolute difference between venous blood samples and VAMS vs. the levels in venous blood samples is shown. No significant trend was observed for any experiment, suggesting that the observed deviations are randomly distributed and not concentration-dependent. On average, Cd and Pb levels in VAMS samples were 0.04 μg/l and 0.31 μg/l, respectively, higher than the corresponding Cd and Pb levels in venous blood. The very low bias is again an indication for proper background compensation using mean background levels. For Pb, the scattering of the differences seems to be less pronounced in pre-cleaned VAMS compared to untreated VAMS, indicating a reduction of uncertainty due to the cleaning process.

### Storage stability of VAMS samples

Storage stability was assessed by a linear regression model (storage time ~ recovery). No statistically significant correlation (*α* = 0.05) was observed between the time stored and the recovery rate for both Cd and Pb as well as for both experiments. This suggests that VAMS samples intended for Cd and Pb analysis in blood are stable for at least seven weeks at ambient conditions.

### Strengths and limitations of the study

This study confirms the applicability of VAMS in human biomonitoring for the simultaneous analysis of Cd and Pb in blood compared to venipuncture as a gold standard under realistic sampling conditions. The sensitivity for Pb was improved by pre-cleaning VAMS, and it was thus possible detect levels in low-exposed individuals. Furthermore, this study contributes towards a wider applicability of this methodology, which promises convenient sample collection, higher acceptability and compliance of participants, and lower logistical costs. Additionally, pre-cleaning was used to overcome one of the most significant challenges of this method, i.e., the risk of high and unevenly distributed background levels. By collecting samples in triplicate and removing outliers by applying a predefined threshold, this led to a significant improvement of the performance. This is particularly relevant since VAMS analysis cannot be repeated after a sample has been extracted. Furthermore, it was demonstrated that using mean background correction is a valid option for VAMS.

A major limitation of the study was the high and unstable background contamination in combination with low levels of Cd and Pb due to an unexposed study population. Subsequently, most of the samples were below the calculated LOQ for VAMS, especially when the sampling devices were used as provided by the manufacturer. While this was overcome for Pb by precleaning VAMS, Cd levels were still mainly below the LOQ in this experiment. Thus, a high uncertainty was observed in this concentration range.

## Conclusions

In conclusion, the performance of VAMS for the analysis of Cd and Pb in blood can be assessed as sufficient, even at low individual exposure. However, the reliability decreases when blood levels of Cd and Pb were lower than the LOQ of VAMS. Nevertheless, extreme overestimations were rare (no more than one sample per experiment), confirming the high reliability of VAMS in general. If this methodology is applied for exposed individuals, e.g., in polluted areas, the issue of interfering background levels will be less prominent and the performance of VAMS is likely to be much better than what has been shown in this study, especially for Cd.

To ensure reliable VAMS results, it is imperative that the samples must be handled with high precaution as even a small contamination, e.g., by dust particles, will interfere with the analysis. VAMS devices are supplied in open containers within a cardboard box, rendering them susceptible to contamination. Contamination issues further continue due to the openly handled, small blood volumes of microsamples, which is especially critical for Cd due to the extremely low concentration in blood of occupationally non-exposed participants. Additionally, VAMS as well as other microsampling devices must be tested for background levels prior to use and, if necessary, cleaned to reduce those levels to an acceptable minimum. It is also recommended to collect at least three VAMS blood samples to allow the identification of outliers, which can significantly impair performance. Due to the small volume, the collection of three or more VAMS samples is not considered a major problem.

In summary, pre-cleaning of VAMS together with careful handling is crucial for a successful application for human biomonitoring of toxic metals.

## Supplementary Information

Below is the link to the electronic supplementary material.

ESM 1 (XLSX 34.4 KB)

ESM 2 (DOCX 160 KB)

## Data Availability

Anonymized Cd and Pb levels in VAMS and venous blood samples as well as in blanks can be found in the supplementary information.
