# Supplementary material for: Application of volumetric absorptive microsampling (VAMS) for the simultaneous determination of cadmium and lead in blood
Source: Environ Monit Assess. 2026 Sep 1;198(9):1018. doi: 10.1007/s10661-026-15796-y (PMC13534157; doi:10.1007/s10661-026-15796-y)
Supplement: Supplementary file 1 [file 10661_2026_15796_MOESM2_ESM.docx]

Figure S1: Box-whisker-plots of blanks results for Cd and Pb for untreated and pre-cleaned VAMS before the exclusion of extreme values.
